# Statin Use Reduces the Risk of Hepatocellular Carcinoma: An Updated Meta-Analysis and Systematic Review

**DOI:** 10.7759/cureus.27032

**Published:** 2022-07-19

**Authors:** Shrouq Khazaaleh, Muhammad Talal Sarmini, Mohammad Alomari, Laith Al Momani, Bara El Kurdi, Mohammad Asfari, Zain Almomani, Carlos Romero-Marrero

**Affiliations:** 1 Internal Medicine, Cleveland Clinic Fairview Hospital, Cleveland, USA; 2 Gastroenterology and Hepatology, Cleveland Clinic Foundation, Cleveland, USA; 3 Gastroenterology and Hepatology, Cleveland Clinic Florida, Weston, USA; 4 Internal Medicine, University of Missouri at Kansas City, Kansas City, USA; 5 Gastroenterology and Hepatology, University of Texas Health Science Center at San Antonio, San Antonio, USA; 6 Internal Medicine, Jordan University of Science and Technology, Alramtha, JOR

**Keywords:** viral hepatitis b and c, cirrhosis, cancer prevention, hepatocellular carcinoma (hcc), statin use

## Abstract

Hepatocellular carcinoma (HCC) is the most common primary tumor of the liver resulting in approximately 800,000 deaths annually. A growing body of research investigating statin use and HCC risk has shown conflicting results. We aim to evaluate the current evidence of statin impact on HCC risk.

We performed a comprehensive literature search in PubMed, PubMed Central, Embase, and ScienceDirect databases from inception through May 2019 to identify all studies that evaluated the association between statin use and HCC. We included studies that presented an odds ratio (OR) with a 95% confidence interval (CI) or presented data sufficient to calculate the OR with a 95% CI. Statistical analysis was performed using the Comprehensive Meta-Analysis (CMA), Version 3 software, and a Forrest plot was generated. We assessed for publication bias using conventional techniques.

Twenty studies (three randomized controlled trials, six cohorts, and 11 case-controls) with 2,668,497 patients including 24,341 cases of HCC were included in the meta-analysis. Our findings indicate a significant risk reduction of HCC among all statin users with a pooled odds ratio of 0.573 (95% CI: 0.491-0.668, I2= 86.57%) compared to non-users. No publication bias was found using Egger’s regression test or on visual inspection of the generated Funnel plot.

The results indicate that statin use was associated with a 43% lower risk of HCC compared to statin non-users. Further prospective randomized research is needed to confirm the association.

## Introduction and background

Hepatocellular carcinoma (HCC) is the most common primary tumor of the liver resulting in approximately 800,000 deaths around the world every year [1]. In many parts of the world, including the United States (USA), both HCC incidence and death rates are increasing [2,3]. The primary liver malignancy occurs most often in regions where Hepatitis B virus (HBV) infection is endemic and is transmitted vertically during birth: Eastern and Southern Asia, Middle and Western Africa, and Polynesia [4]. For reasons not fully understood, HCC is more common in males than females, 6.8 per 100,000 persons vs. 2.2 per 100,000 persons, respectively in North America (NA) [5]. With HCC being the fourth leading cause of cancer-related deaths in the world, the financial burden of treatment is significant [2]. In the USA alone, the mean three-year cost of care for each patient is estimated to be around $154,688 [6]. With the increasing incidence of cirrhosis and HCC, combined with growing health care costs, understanding how statin use is associated with a lower risk of HCC is critical.

HCC results from states of chronic liver inflammation, which cause free radical release, DNA damage, gene mutations, and alterations in the ability of hepatocytes to repair damaged DNA [7-9]. As HCC is often diagnosed late in the course of the disease, numerous studies have directed their focus toward reducing the risk of developing HCC through various protective factors [10]. The most documented protective factors include vaccination against HBV and antiviral treatment of both HBV and hepatitis C virus (HCV). In addition, some diabetes mellitus (DM) medications (i.e. metformin and pioglitazone), as well as coffee, were found to be protective against HCC formation. In the past two decades, significant research has been conducted on statin use and HCC incidence [11-14].

Statins are 3-hydroxy-3-methylglutaryl coenzyme A reductase inhibitors that lower lipid levels; therefore, their role in cardiovascular disease treatment and prophylaxis is essential [15]. Besides the effect of cholesterol formation, statins are known to have proapoptotic, antiangiogenic, antiproliferative, and immunomodulatory effects. For these reasons, many researchers have aimed to assess whether statins can decrease the risk for multiple malignancies, including HCC [11-14].

The results of observational and randomized controlled trials (RCTs) have not been consistent. While most observational studies showed statistically reduced risk for HCC in patients who were using statins, the data from the RCTs did not. Hence, we conducted this study to examine the association between statin use and HCC utilizing all available published high-quality literature.

The preliminary data of this work were presented at the 2019 American College of Gastroenterology (ACG) conference; October 25, 2019/San Antonio-Texas, in the form of podium presentation. The abstract was published in the American Journal of Gastroenterology: October 2019 - Volume 114 - Issue - p S545-S546.

## Review

Methods

Search Strategy and Selection Criteria

We performed a comprehensive literature search in PubMed, PubMed Central, Embase, and ScienceDirect databases from inception through May 31, 2019, to identify all studies that evaluated the association between statin use and new HCC development. Keywords used in our search included: statin(s), risk, “Atorvastatin,” “Rosuvastatin,” “Fluvastatin,” “Lovastatin,” “Pravastatin,” “Simvastatin,” “Pitavastatin,” and “Cerivastatin” combined with one of the following; HCC, liver neoplasm(s), and liver cancer incidence. The search was limited to human studies with no restrictions placed on region, publication type, or language. References of all included studies were manually searched for additional eligible papers.

Data Extraction and Quality Assessment

Two authors independently performed the literature review (M.A and L.A). The data from the included studies were entered into a standardized table for analysis. To be included, studies were required to meet the following criteria: 1) Implemented a well-defined RCT, case-control, or cohort design; and 2) Presented an odds ratio (OR) for our main outcome with a 95% confidence interval (CI) or presented the data sufficient to calculate the OR with a 95% CI. Studies were excluded if they provided insufficient information to calculate the OR for our main outcome. Studies were excluded if they were letters to authors, case reports, case series, or review articles.

The quality of included studies was assessed independently by two of the authors (S.K. and M.A.) using the Newcastle-Ottawa Scale and the Jadad scale for observational studies and randomized clinical trials, respectively. L.A. and M.S. addressed the discrepancies by joint evaluation of the original article [16,17].

Statistical analysis

Statistical analysis was performed using the Comprehensive Meta-Analysis (CMA), Version 3 software (BioStat, Inc., Englewood, NJ). Effect estimates from the individual studies were extracted and combined using the random-effect, generic inverse variance method of DerSimonian and Laird [18]. A random effect model was used as a high probability of between-study variance was suspected due to variation in study population and methodology. A pooled OR was calculated. A Cochran's Q-test and an I2 statistic were used to evaluate heterogeneity and quantify variation across the selected studies [19]. A funnel plot was then created to evaluate for publication and other reporting biases and then the plot was examined visually for asymmetry. Then, an Egger test for asymmetry of a funnel plot was conducted. All authors had access to the study data and reviewed and approved the final manuscript.

Results

Search Results

**Figure 1:**
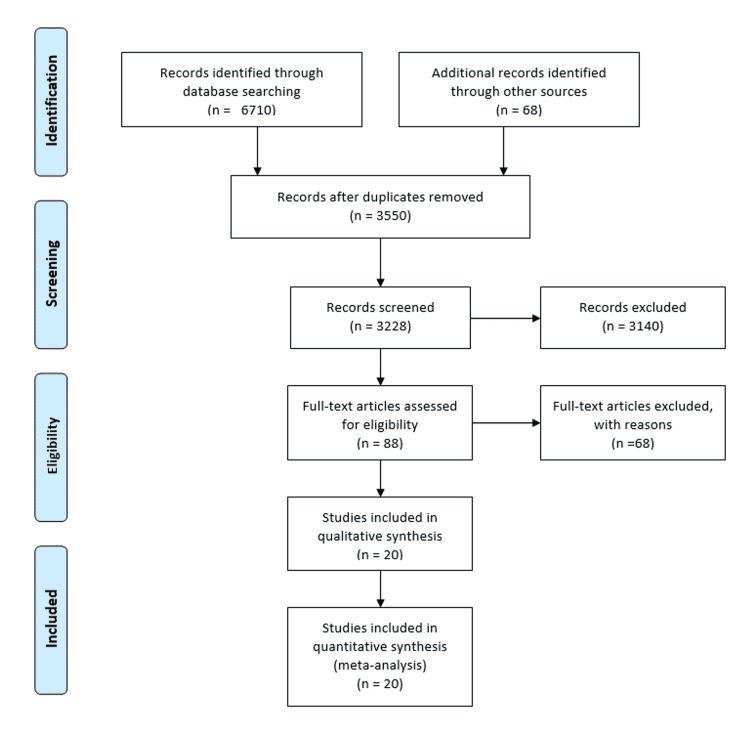
PRISMA flowchart illustrating the selection process.

Our initial comprehensive search yielded 6,710 citations. All citations underwent a title and abstract review, with the majority being excluded for either being letters to authors, case reports, or case series. Of our initial yield, 88 citations underwent a full-length article review, and 68 were excluded as they did not include controls, were review articles, or did not provide sufficient information to calculate the OR for our main outcome. A PRISMA flowchart illustrates the selection process, Figure 1. Consequently, a total of 20 studies (three RCTs, six cohorts, and 11 case-controls) met our inclusion criteria and were included in the meta-analysis [11,20-37]. Baseline characteristics of the included studies and patients are summarized in Tables 1, 2, respectively.

**Table 1:** Baseline characteristics of the included studies * A study can be awarded a maximum of one star for each numbered item within the Selection and Exposure categories. A maximum of two stars can be given for Comparability. Scored items listed below: -  Representativeness of the exposed cohort -  Selection of the non-exposed cohort -  Ascertainment of exposure -  Demonstration that outcome of interest was not present at the beginning of the study -  Cohort comparability based on design -  Assessment of outcome -  Was follow-up long enough for outcomes to occur -  Follow-up adequacy in terms of completeness

Study	Design	Location	Setting	Time period	No. of subjects	No. of HCC cases	Study Quality		
							Selection (Randomized)	Comparability (Double-blind)	Outcome/exposure (Withdrawals)
Tsan et al. [11]	Cohort	Taiwan	Patients with HBV	1997-2008	33, 413	1,021	***	**	***
Chiu et al. [20]	Case-control	Taiwan	Population-based	2005-2008	2,332	1,166	***	**	**
Friis et al. [21]	Cohort	Denmark	Population-based	1989-2002	334,754	171	****	*	***
Marelli et al. [22]	Cohort	USA	Population-based	1991-2009	91,714	105	****	**	***
Friedman et al. [23]	Cohort	USA	Population-based	1994-2003	361,859	42	****	–	**
Khurana et al. [24]	Case-control	USA	Population-based	1997-2002	480,306	409	*	*	–
McGlynn et al. [25]	Case-control	UK	Population-based	1988-2011	5,835	1,195	****	*	**
Bergman et al. [26]	Case-control	Sweden	Population-based	2006-2010	23,964	3,994	****	*	**
Lai et al. [27]	Case-control	Taiwan	Population-based	2000-2009	17,400	3,480	****	**	**
El-Serag et. al [28]	Case-control	USA	Patients with DM	2001-2002	6,515	1,303	***	**	***
Hsiang et al. [30]	Cohort	China	Patients with HBV	2000-2012	53,513	6,883	****	**	**
Kim et al. [31]	Case-control	Korea	Population-based	2002-2013	514,866	1,642	****	**	**
Simon et al. [32]	Case-control	USA	Patients with HCV	2001-2014	9,135	239	****	**	**
Chen et al. [33]	Case-control	China	Population-based	2000-2010	34,672	340	****	**	**
Chen et al. [34]	Case-control	China	Patients with HBV	2000-2008	71,824	1,735	***	**	**
Tran et al./PCCIU [35]	Case-control	UK	Population-based	1999-2011	2103	434	****	**	***
Tran et al./UK Biobank [35]	Prospective cohort	UK	Population-based	2006-2010	475,768	182	***	**	**
Matsushita et al. [36]	RCT	Japan	Individual patient data analysis of trials	2010	13,724	12	(N/A)	(N/A)	(N/A)
CTT [37]	RCT	Europe, Australia, NA	Individual patient data analysis of RCT	2012	134,537	68	(N/A)	(N/A)	(N/A)
Sato et al. [38]	RCT	Japan	Secondary analysis of RCT	1991-1995	263	1	(1)	(1)	(–)

 

**Table 2:** Baseline characteristics of the included patients

Study	Age (y)		Sex (% male)		Cirrhosis (% total)		HBV/HCV (% total)	
	Case	Control	Case	Control	Case	Control	Case	Control
Tsan et al. [11]	34.7	46.3	57.1	58.3	11.6	10.6	100/0	100/0
Chiu et al. [20]	66.1	65.9	68.9	68.9	39.4	4.9	23.9/25.1	5.3/3.5
El-Serag et al. [28]	72	72	99	99	28.2	1.6	1.9/14.7	0.2/1.8
Friis et al. [21]	60.7	46.6	57	50	NA		NA	
Marelli et al. [22]	64.2	64.2	52.2	52.6	NA		0.06	0.07
Friedman et al. [23]	NA		NA		NA		NA	
Khurana et al. [24]	61.1		91.7		NA		NA/2.9	
Matsushita et al. [36]	57.9	57.1	52.6	50.5	NA		NA	
CTT [37]	63		71		NA		NA	
Sato et al. [38]	NA		81.7		NA		NA	
McGlynn et al. [25]	67.2	67	71.6	71.6	NA		NA	
Bergman et al. [26]	NA	NA	52	52	NA		NA	
Lai et al. [27]	62.7	62	72.6	72.6	52.4	1.29	37.2/28.9	3.05/1.97
Hsiang et al. [30]	58.7	37.6	67.9	25.5	2.7	1.6	100/0	100/0
Kim et al. [31]	61.8	61.8	83.6	83.6	34.2	1.1	NA	
Simon et al. [32]	53.5	52.5	96.16	95.37	14.02	21.43	0/100	0/100
Chen et al. [33]	62.6	62.4	77.9	77.9	NA		41.8/31.5	5.8/3.3
Chen et al. [34]	NA		55	57	NA		100/0	100/0
Tran et al./PCCIU [35]	NA		67	67	NA		NA	
Tran et al./UK Biobank [35]	NA		62	46	NA		NA	

Characteristics of Included Studies

The characteristics of the studies used in the meta-analysis are shown in Table 1. Three studies were RCTs. Sato et al. conducted the first RCT of pravastatin use with HCC risk in 2006 and included 263 patients admitted to the Osaka Medical Center for Cancer and Cardiovascular Diseases between 1991 and 1995 [37]. Matsushita et al. conducted the second RCT in 2010 to study the association between pravastatin use and risk of cancer including 13,724 patients in total and 12 patients with HCC [36]. Conducted in Japan like Sato et al., Matsushita et al. analyzed patient data collected from three large-scale prospective studies: the Management of Elevated Cholesterol in the Primary Prevention Group of Adult Japanese Study, Kyushu Lipid Intervention Study, and Hokuriku Lipid Coronary Heart Disease Study-Pravastatin Atherosclerosis Trial. The third and most recent RCT studying the association between statin use and risk of cancer published in 2012, called the CTT Collaboration trial, included a total of 134,537 patients and 68 patients with HCC [37]. It was noted that 58% of the patients were on hydrophilic statins (pravastatin and rosuvastatin).

Six cohort studies have reviewed the association between statins and HCC risk [11,21-23,29,34]. The Friis et al. study published in 2005 included 334,754 patients from the Prescription Database of North Jutland County and the Danish Cancer Registry for the period 1989-2002 [23]. Tsan et al., Hsiang et al., and Tran et al./UK Biobank each included large populations of 1,021, 6,883, and 182 patients with HCC, respectively [11,29,34]. Tsan et al. studied 260,864 HBV-infected patients enrolled in the Taiwan National Health Insurance Research Database between 1999 and 2010. Hsiang et al. utilized the Hospital Authority database in Hong Kong to collect their data, and in their two studies, Tran et al./PCCIU and Tran et al./US Biobank, performed a nested case-control study within the Scottish Primary Care Clinical Informatics Unit (PCCIU) database, and a prospective cohort study within the UK Biobank, respectively. Friedman et al. is a 2007 cohort study that recorded receipt of statins in subscribers of Kaiser Permanente Medical Care Program in northern California [23]. They collected their data from the program’s pharmacy records and cancer registry from 1994 to 2003. Their cohort included 361,859 statin users. Marelli et al. is a 2011 retrospective cohort analysis of the incidence of cancer in older adults who have and have not used statins [22]. The study was performed from the General Electric Centricity electronic medical records database. Propensity score methods matched 45,857 comparison pairs of statin and non-statin users. Chen et al. conducted a 2015 cohort study using the Taiwan Longitudinal Health Insurance Database between 2000 and 2008 [33]. This cohort study comprised 71,824 HBV-infected patients.

Eleven case control studies were included in the analysis. Published in 2005, Khurana et al. included 480,306 patients, 409 with HCC [24]. It is the second largest case-control study on the association between statin use and risk of HCC. The largest case control study included in the meta-analysis was published in 2018 by Kim et al., which utilized data from the National Health Insurance Service Physical Health Examination Cohort between 2002 and 2013 in Korea [20,24-28,30-33]. The two studies conducted on the relationship between statin use and risk of HCC with the largest population of patients with HCC were conducted in 2013 and 2014 by Lai et al. and Björkhem-Bergman et al., respectively [26,27]. Lai et al. utilized the Taiwan National Health Insurance program to conduct a case-control study with 3,480 patients with newly-diagnosed HCC identified between 2000 and 2009. Björkhem-Bergman et al. utilized the Swedish Cancer Register and the Swedish Prescribed Drug Register to conduct a case-control study with 3,994 patients with newly-diagnosed HCC identified between 2006 and 2010. Chiu et al. conducted a population-based case-control study in Taiwan in 2010 using data collected from the Taiwan National Health Insurance Research Database of patients with a first-time diagnosis of liver cancer for the period between 2005 and 2008 [20]. Chiu et al. examined 1,166 liver cancer cases and 1,166 controls. Simon et al. and El-Serag et al. are both case-control studies of USA Veteran patients [28,31]. Simon et al. used the Electronically Retrieved Cohort of HCV Infected Veterans (ERCHIVES) database in 2016 to identify patients initiated on HCV therapy from 2001 to 2014, and all incident cases of cirrhosis and HCC. El-Serag et al. was a 2009 matched case-control study nested within a cohort of patients with diabetes during the calendar years 1997-2002 in the Department of Veterans Affairs (VA) national databases. El-Serag et al. examined 1,303 cases and 5,212 controls.

Other studies include nested case-control studies completed by McGlynn et al. and Chen et al. [25,32]. McGlynn et al. is a 2014 nested case-control study conducted among members of the Health Alliance Plan HMO of the Henry Ford Health System enrolled between 1999 and 2010. Electronic pharmacy records of statin use were compared among 94 tumor registry-confirmed cases of HCC versus 468 controls. Chen et al. is a 2015 nested-case-control study referencing the Longitudinal Health Insurance Database established by the Taiwan Bureau of National Health Insurance. The cohort in this study comprised 340 patients with HCC and 1,360 controls.

Quality of Included Studies

The median Newcastle-Ottawa quality score for 17 observational studies was 8 (range: 2-9) and 15 (88%) had a cumulative score of at least 7, thus were considered methodologically high-quality studies (Table 1). The cumulative Jadad scale score was 2 for one study, whereas the other two studies analyzed individual data from multiple RCTs and the scale was not applicable to assess the randomization strategy.

Meta-analysis Results

**Figure 2:**
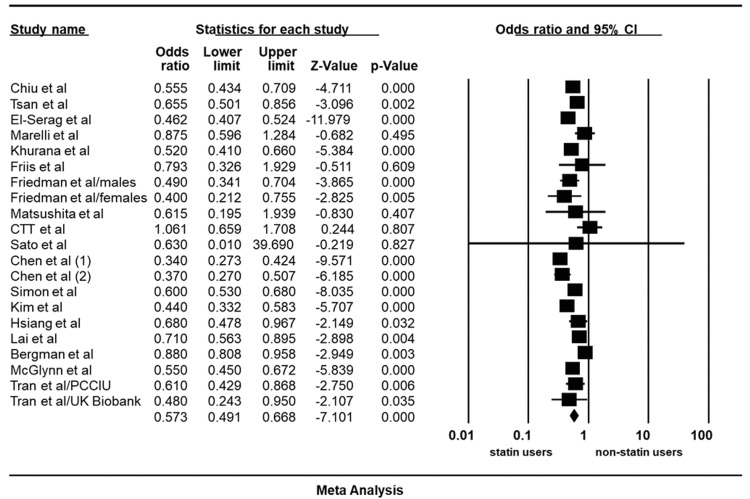
Summary of odd ratios assessing the incident hepatocellular carcinoma with statin use CI: Confidence interval Tsan et al. [11], Chiu et al. [20], Friis et al. [21], Marelli et al. [22], Friedman et al. [23], Khurana et al. [24], McGlynn et al. [25], Bergman et al. [26], Lai et al. [27], El-Serag et al. [28], Hsiang et al. [30], Kim et al. [31], Simon et al. [32], Chen et al. (1) [33], Chen et al. (2) [34], Tran et al./PCCIU [35], Tran et al./UK Biobank [35], Matsushita et al. [36], CTT [37], Sato et al. [38].

Risk reduction among statin users: Twenty studies (three RCTs, six cohorts, and 11 case-controls) met our inclusion criteria and were included in the meta-analysis [11,20-37]. These studies include a total of 2,668,497 patients. Of these patients, 24,341 had HCC. Five of the studies were conducted in the USA, six in Europe (Denmark, Sweden, United Kingdom), and nine in Asia (China, Japan, South Korea, Taiwan). Our findings indicate a significant risk reduction of HCC among all statin users with a pooled odd ratio of 0.573 (95% CI: 0.491-0.668, p<0.05, I2= 86.57%) compared to non-users (Figure 2).

Evaluation for publication bias: A Funnel plot was generated to evaluate the risk reduction of HCC among all statin users. The plot is symmetric and does not suggest the presence of publication bias. Egger’s regression asymmetry testing was also done to demonstrate no evidence of publication bias (p>0.05).

Subgroup and sensitivity analysis

We performed subgroup analyses of the 21 cohorts in 20 studies based on study location and design (Table 3).

**Table 3:** Subgroup and sensitivity analysis

Subgroup Analysis	No of cohorts	No of patients	Adjusted HR (95% CI)	Test of heterogeneity I^2 ^(%), P-value		Heterogeneity between groups, P-value
Study location						0.19
Asia	9		0.518 (0.414 - 0.647)	75	<0.0001	
Europe	6		0.707 (0.538 - 0.929)	79	0.0002	
USA	6		0.544 (0.459 - 0.646)	68	0.007	
Study design						0.018
Observational	18		0.557 (0.475 - 0.652)	88	<0.0001	
RCT	3		0.975 (0.629 - 1.509)	0	0.68	
Sensitivity analysis to determine source of heterogeneity in observational studies						
Study quality						0.31
High quality	15		0.572 (0.479 - 0.682)	90	<0.0001	
Low quality	3		0.500 (0.413 - 0.604)	0	0.74	
Study design						0.26
Case-control	11		0.534 (0.436 - 0.653)	93	<0.0001	
Cohort	7		0.625 (0.520 - 0.752)	23	0.26	

Statin use was associated with 48%, 29%, and 46% risk reductions in studies performed in Asia, Europe, and USA, respectively. Although moderate heterogeneity was seen within the groups. On stratified analysis based on study design, RCTs did not show any statistically significant association between statin use and HCC, without significant heterogeneity within the group. In 18 cohorts of 17 observational studies, statin use was associated with 44% risk reduction in incidence of HCC. However, there was significant heterogeneity within the group.

Additional sensitivity analysis was performed within the observational studies based on study design and the quality of included studies (Table 3). However, this analysis did not further explain the source of heterogeneity in the overall analysis. Fifteen high-quality and three low-quality studies were associated with 43% and 50% risk reductions in incidence of HCC, with no heterogeneity within the groups, respectively. Eleven case-control and seven cohort studies were associated with 47% and 37% risk reductions in incidence of HCC, with no heterogeneity within the groups, respectively.

Meta-regression analyses

To further evaluate sources of heterogeneity, we screened the data reported in the studies for patterns which might affect the OR of HCC across different studies. We hypothesized that the statin dose might affect this protective effect. We chose the measure of defined daily dose (DDD), a unit recommended by the World Health Organization, which is the assumed average maintenance dose per day of a drug consumed for its main indication. DDD was used to reflect dosing in order to reduce within-person variation and better estimate long-term intake of the medication. We found that seven studies had reported this measure, we therefore included them in a meta regression model to evaluate the correlation between DDD and the protective effect of statins.

Reported DDDs were pooled and divided into four categories. Each category was given an incremental score from 1 to 4. A logistic regression model was constructed to evaluate the correlation between the proposed scoring system reflecting the relationship between the protective effect of statin and the dose used. DDD < 180 was given a score of 1, DDD > 180 was given a score of 2, DDD < 360 was given a score of 3 and DDD > 360 was given a score of 4.

**Figure 3:**
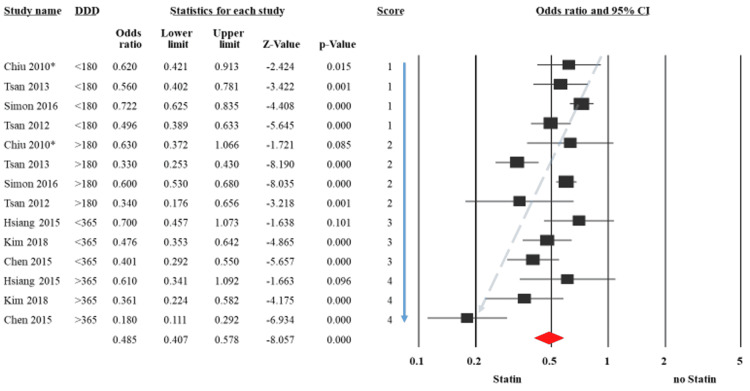
Summary of odd ratios assessing the incident of hepatocellular carcinoma based on DDD of statin use. DDD: Defined Daily Dose Tsan et al. [11], Chiu et al. [20], Hsiang et al. [30], Kim et al. [31], Simon et al. [32], Chen et al. [33].

**Figure 4:**
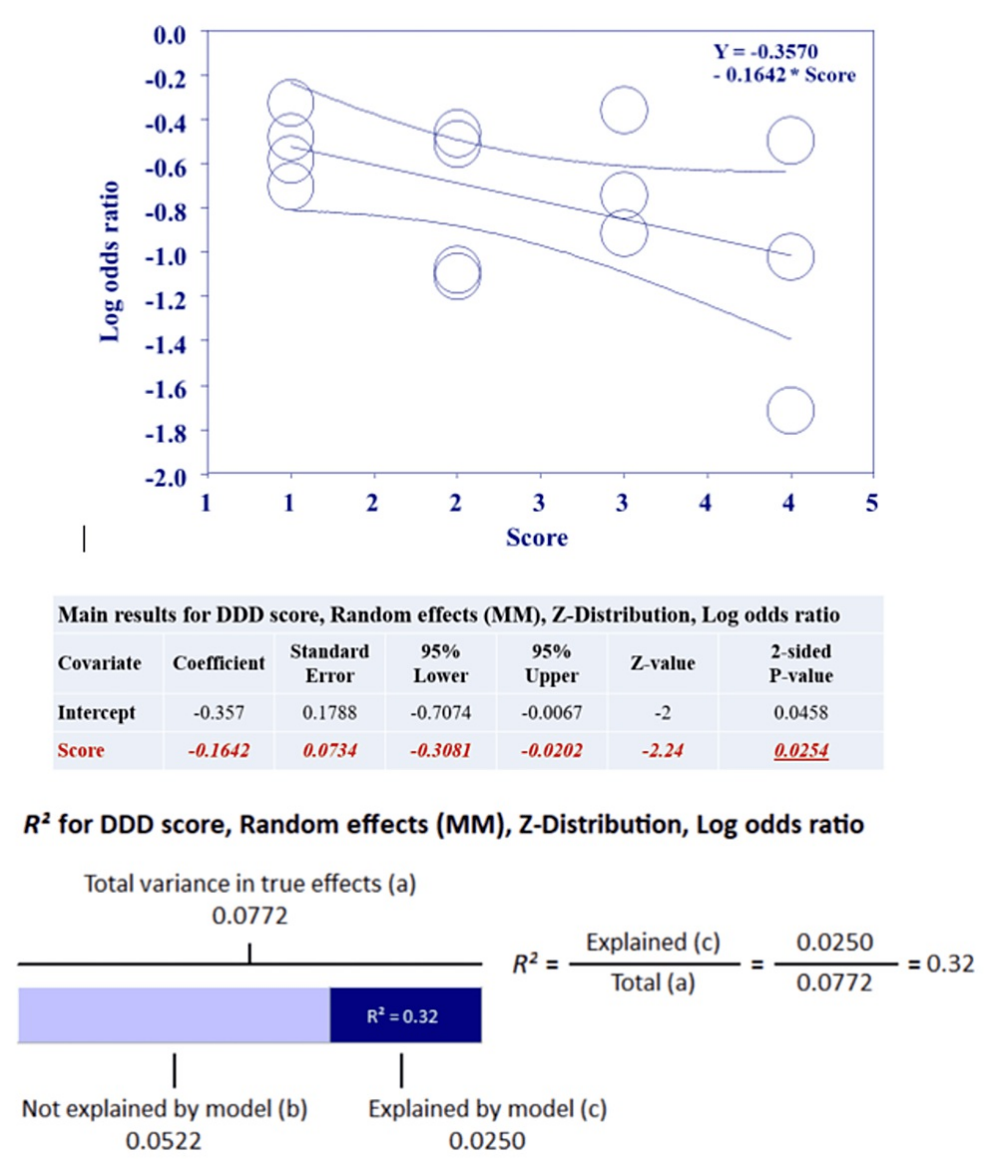
A random-effects model showing regression of log odd ratio on score, and proportion of variance explained by model.

Figure 3 shows a Forrest plot of OR of HCC for statin users vs. non-users sorted according to the DDD score. Figure 4 represents a visual depiction of the logistic regression results showing a negative correlation between the DDD score and the OR for HCC. Figure 4 illustrates the statistical results showing a significant correlation between the DDD score and the OR for HCC. In addition, it shows that this model was able to explain 32% of the heterogeneity noted in the OR between the seven studies included in this sub-analysis.

Discussion

In the current investigation, we found a 43% risk reduction of HCC among statin users compared to non-users. Our meta-analysis includes three RCTs: Sato et al., Matsushita et al., and the CTT trial, published in 2006, 2010, and 2012, respectively [35-37]. The three RCTs are in concordance in their conclusions that statin use does not affect the rate of cancer incidence. Unfortunately, interpretation of these results in the context of HCC risk is difficult because of the small incidence rate of this disease in the three studies. Moreover, these studies were not primarily aiming to measure HCC occurrence; therefore, patient screening may have been less than ideal for identifying HCC. Though contextually important, these studies accounted for a small minority of the included HCC cases (81 cases; 0.3%).

Statins are well-established as both safe and efficacious in the prevention of cardiovascular disease. Recent studies have demonstrated that statins may also work to lower risk of some cancers and ageing-related diseases; however, the data are neither strong nor consistent [38]. More recently, researchers have focused their efforts towards investigating the preventative effect of statins on HCC. A considerable but variable body of research has been put forward to evaluate the association between statin use and HCC. For this reason, the present meta-analysis aimed to examine the previously published studies on this association.

Six cohort studies reviewed the association between statin use and HCC risk and were included in our meta-analysis [11,21-23,29,34]. Four cohort studies found significant lower HCC risk, while two did not. Friis et al. was the first to find that individuals prescribed statins experienced a slightly reduced cancer incidence compared to population controls of non-users and users of other lipid-lowering drugs [21]. Cohort studies by Tsan et al., Hsiang et al., and Tran et al./UK Biobank have found a consistent inverse relationship between statin-use and the risk of primary liver cancer [11,29,34]. This result was seen specifically in patients with HCC, and research included large populations of patients with HCC. Eleven case-control studies have reviewed the relationship between statin use and the risk of HCC, including the largest case study published in 2018 by Kim et al., which concluded that statin use may have beneficial inhibitory effects on HCC risk [20,24-28,30-33]. Despite consistently demonstrating a reduced risk of HCC in statin users, the results in these observational studies must be cautiously interpreted. That is because the case-control and cohort studies lack the random allocation of interventions that allow the RCTs to test their hypotheses. Eliminating confounding of data in observational studies is impossible, and the data must be understood in this context.

While the observational data demonstrate the reduced risk of HCC in statin users, the mechanism by which this protective effect occurs is unknown. In the past decade, several hypotheses have been offered to explain this phenomenon. Statins are well known to inhibit cholesterol synthesis by inhibiting the production of mevalonate. Some studies hypothesize that the reduction in mevalonate may cause a decrease in the growth and proliferation of neoplastic cells, explaining their ability to reduce HCC risk [39]. The inhibition of the mevalonate pathway may have downstream effects, not only including the disruption of growth of malignant cells but also increasing apoptosis [40]. This is perhaps the result of regulating the MAPK/ERK pathway and possibly other signaling pathways [39]. Alternatively, statins may be targeting ubiquinone in hepatocytes causing the apoptosis of pre-neoplastic cells [39]. Reduction in mevalonate may cause a decrease in geranylgeranyl pyrophosphate levels and lead to inhibition of HCV viral RNA replication. Thus, statins may reduce HCC risk by exerting an anti-HCV effect [41,42]. Thirdly, statins are also known to inhibit inflammation and angiogenesis, and studies have suggested that statin use might lead to a decrease in carcinogenesis because of their anti-inflammatory and anti-angiogenetic effects [39,42,43]. In addition, lower cholesterol level is independently inversely related to risk of liver cancer [39,44,45].

Several studies have found reduction in HCC risk to be dependent on the statin dose [11,28,30,31,33,34]. The HCC risk reduction varied substantially by study [11,21,28-31,33,34]. Chen et al. found that middle to high cumulative defined daily doses (cDDDs) of a statin was necessary to have a reduced risk of liver cancer (middle: 91-365 cDDDs, high: >365 cDDDs) [33]. Kim et al. also found a dose dependent effect with the greatest risk reduction in statin users with doses greater than 720 cDDDs [30]. Our meta regression results were in line with the abovementioned findings. As DDD increased, the chances of HCC development dropped significantly. This could indicate that higher doses might infer stronger protection. While statin dosing has long-been stratified into low, moderate, and high-intensity for cardiovascular preventive purposes, further research is needed to adequately stratify the dose-effect relationship for statins’ HCC preventive qualities.

Our analysis shows that the statistical heterogeneity of our study was moderate. We excluded one study at a time to observe its individual effect on the pooled OR, which remained approximately the same. Our subgroups analysis suggested that patients on statins in Asia and the USA had a more profound HCC reduction rate compared to patients from Europe. In addition, assessing three studies that evaluated HBV patients, we found that HBV patients on statins had 45% less risk of developing HCC. After applying the Newcastle-Ottawa scale to evaluate the quality of the observational studies, patients in high quality studies still had a statistically significant lower OR for HCC. Finally, our meta regression model was able to explain 32% of the heterogeneity noted in OR among the seven studies included in this sub-analysis. Although this finding cannot be generalized to all included studies, it remains highly suggestive that other studies with unreported DDDs have similar differences in statin dosing that contributed to the observed significant heterogeneity in this meta-analysis.

A major limitation of our study is the inconsistency of controlling for confounders among the individual studies included. Most studies failed to control for one or more risk factor for HCC including alcoholic liver disease, nonalcoholic steatohepatitis (NASH), DM, HBV, HCV, and cirrhosis. Furthermore, studies failed to control for the use of concomitant medications, such as metformin and thiazolidinediones, which have been associated with reduced HCC risk [46,47]. Additionally, the duration and dosage details of the statin therapies was not available for most of the studies. Although we included studies from Asia, Europe, and NA, our subgroup analysis showed that statins had statistically significant lower odds for HCC in individuals included separately, which likely eliminates the role for geographical bias. The lack of data from other countries and races could limit the extrapolation of results to other demographics. Finally, the risk reduction of HCC among statin users compared to non-users demonstrated in this meta-analysis must be weighted in the context of potential adverse effects, access, and cost of long-term therapy.

One area of potential future research includes statin type and their impact on HCC risk. El-Serag et al. found a reduced trend was similar for all six statins included in the study, a 25%-40% reduction, despite theories that lipophilic statins (such as simvastatin, atorvastatin, and fluvastatin) are more liver-specific, and therefore, have larger HCC risk reduction compared to hydrophilic statins (pravastatin and rosuvastatin) [37,48]. However, other studies found a significant difference in reduction in HCC risk with statin type, with pravastatin specifically not having any significant difference on HCC risk [27,32,34,35,37].

In 2016, analyses by Zhou et al. found fluvastatin to be the most effective statin for reducing HCC risk compared with other statins, while rosuvastatin was the least effective in reducing HCC rate [49]. Unfortunately, subtype group size was particularly small after being divided into different types of statins in Zhou et al., limiting their meta-analysis. Following Zhou et al., Kim et al. found that both hydrophilic and hydrophobic statins showed significant beneficial effects [30]. In 2019, Tran et al./PCCIU found only simvastatin was significantly associated with reduced liver cancer risk [34]. Further prospective RCTs comparing individual statins are needed to show what differences in the beneficial effects HCC risk exist among different statins as previous trials have had conflicting results [27,28,32,35]. The three RCTs included in this meta-analysis had few patients at high-risk for HCC. They had relatively short follow up, and most patients were on hydrophilic statins which are theorized to be less effective.

## Conclusions

In summary, this meta-analysis expands on a body of literature published on the association between statin use and reduced HCC risk. Our research includes twenty studies of various types, totals over 2.6 million individuals from Asia, Europe, and North America, and employs the most current analytical techniques to provide important data for clinicians and researchers alike. We found that statin use was associated with a 43% lower risk of HCC. However, the results should be interpreted with caution given the possibility of uncalculated cofounding factors. Although further prospective randomized research is needed to confirm this association and to understand the underlying pathophysiology, statin use might be considered in patients at high risk for HCC, particularly in the presence of other indications.
